# The Conveyance of Infection

**Published:** 1897-04-03

**Authors:** 


### The Hospital

A Journal of

The Medical Sciences and Hospital Administration.

Vol. XXII.—No. 549.] [April 3, 1897.

### The Conveyance of Infection.

The frequent conveyance of infectious disease from place to place, from person to person, and from community to community, by non-living materials of various kinds which had been in contact with the bodies of the sick, or with emanations from those bodies, must have forced itself upon the attention of mankind at a very early period of human history. The various possible carriers of such infection were described generally by the physicians of a former age as "fomites" (*fomes*—tinder); and bedclothes, bedding, woollen garments, carpets, curtains, and letters were enumerated as the most important of them. The "fomes" by which the plague of 1665 was taken from London to the Derbyshire village of Eyam, where, in the course of thirteen months, it destroyed 260 lives out of a population of 350, was said at the time to be a parcel of tailors' patterns; and it cannot be any matter for surprise that, not only in the seventeenth century, but before its commencement and for long after its close, all the common "fomites" were regarded with equal apprehension, whatever might be the nature of any disease which prevailed in the locality from whence they had proceeded. In the days when quarantine was almost universal, the hapless passengers arriving from an infected country, or from one in which any dangerous form of communicable disease existed, were themselves liable to be smoked and fumigated in all manner of ways, while not only was the wearing apparel in their trunks dealt with in a similar and usually in a practically destructive manner, but letters, whether carried among baggage or in the mail bags, were made the subjects of very particular attention, and were partially cut open, or perforated in many directions, in order that some supposed "disinfectant" of a volatile character might obtain free access to their inmost folds. The possible or probable obliteration of the writing was not a matter which would weigh at all seriously upon the mind of the average health-officer of a continental port.

Apart from accidental errors, no one ever sees any disease produced by the contagion of any other, but each one, to use a common form of expression, "breeds true" to its own special characteristics. It follows that, in each case, the living agent of infection, now assumed to be almost invariably a microbe belonging to the vegetable kingdom, must have its own individual life history, different from that of other microbes, its allies; and that, while certain modes of transmission, or certain "fomites," may provide for the conveyance of one form in full activity, they may be injurious or even fatal to others. In order, therefore, to know how any particular disease is likely to be transmitted, and by what particular precautions its transmission may most effectually be guarded against, it is necessary to know the behaviour of its microbe, the circumstances by which that microbe is enabled to multiply in customary or even in enhanced virulence, as well as those by which its powers for evil may be attenuated or destroyed. By the aid of such knowledge it becomes possible, in regard to each variety of contagion, to guard against the particular fomites by which it is most liable to be conveyed, and to neglect others which may not be sources of danger in the particular instance. We know, for example, that the microbes of enteric fever and of cholera are present in a state of dangerous activity in the intestinal discharges of the sick, and that they multiply exceedingly, without loss of virulence, when the discharges containing them find access to water under certain conditions. There is much reason to believe that they do not leave the bodies of patients through any other channel, and hence that, if the infection of water by intestinal discharges, including, of course, any which may have found their way to bedding or clothing, be prevented with sufficient care, the disease will not spread, and all "fomites" other than those contaminated by intestinal discharges may be safely neglected. In diphtheria, as in pulmonary tuberculosis, the chief danger probably resides in the secretions of the respiratory mucous membrane; and the conveyance of the microbe seems constantly to be effected by the pulverisation of such secretions when they are dry, and by their subsequ[illegible]ribution through the medium of the atmosphere, to be breathed or swallowed by healthy persons. In such cases, therefore, soiled pocket-handkerchiefs are the

"fomites" most carefully to be disinfected and guarded against. In anthrax, again, to pass from human maladies to one of which the chief stress falls upon the brute creation, the experiments of Pasteur have shown that moist earth is the favourite breeding ground of the microbe, and that it will be brought up by earthworms from the carcasses of sheep which were buried in the locality ong before. The general result is always the same; namely, that, in order to guard against the conveyance of infection, with the smallest amount of suffering to the sick, and with the smallest amount of inconvenience to the healthy, it is necessary to know the life history of each form of infective material, the particular fomites in which it is most liable to be conveyed, and the conditions which are respectively favourable or detrimental to its activity.

